# Electroacupuncture Zusanli (ST36) Relieves Somatic Pain in Colitis Rats by Inhibiting Dorsal Root Ganglion Sympathetic-Sensory Coupling and Neurogenic Inflammation

**DOI:** 10.1155/2023/9303419

**Published:** 2023-03-02

**Authors:** Yi-li Wang, Hai-yan Zhu, Xi-qian Lv, Xing-ying Ren, Ying-chun Peng, Jin-yu Qu, Xue-fang Shen, Ran Sun, Meng-lu Xiao, Hong Zhang, Zhao-hui Chen, Peng Cong

**Affiliations:** ^1^Chengdu Medical College, Chengdu, 610500 Sichuan, China; ^2^The First Affiliated Hospital of Chengdu Medical College, Chengdu, 610500 Sichuan, China; ^3^Chengdu University of Traditional Chinese Medicine, Chengdu, Sichuan 610075, China

## Abstract

Referred somatic pain triggered by hyperalgesia is common in patients with inflammatory bowel disease (IBD). It was reported that sprouting of sympathetic nerve fibers into the dorsal root ganglion (DGR) and neurogenic inflammation were related to neuropathic pain, the excitability of neurons, and afferents. The purpose of the study was to explore the potential and mechanism of electroacupuncture (EA) at Zusanli (ST36) for the intervention of colon inflammation and hyperalgesia. Sprague-Dawley (SD) was randomly divided into four groups, including control, model, EA, and sham-EA. Our results showed EA treatment significantly attenuated dextran sulfate sodium- (DSS-) induced colorectal lesions and inflammatory cytokine secretion, such as TNF-*α*, IL-1*β*, PGE2, and IL-6. EA also inhibited mechanical and thermal pain hypersensitivities of colitis rats. Importantly, EA effectively abrogated the promotion effect of DSS on ipsilateral lumbar 6 (L6) DRG sympathetic-sensory coupling, manifested as the sprouting of tyrosine hydroxylase- (TH-) positive sympathetic fibers into sensory neurons and colocalization of and calcitonin gene-related peptide (CGRP). Furthermore, EA at Zusanli (ST36) activated neurogenic inflammation, characterized by decreased expression of substance P (SP), hyaluronic acid (HA), bradykinin (BK), and prostacyclin (PGI2) in colitis rat skin tissues corresponding to the L6 DRG. Mechanically, EA treatment reduced the activation of the TRPV1/CGRP, ERK, and TLR4 signaling pathways in L6 DRG of colitis rats. Taken together, we presumed that EA treatment improved colon inflammation and hyperalgesia, potentially by suppressing the sprouting of sympathetic nerve fibers into the L6 DGR and neurogenic inflammation via deactivating the TRPV1/CGRP, ERK, and TLR4 signaling pathways.

## 1. Introduction

Inflammatory bowel disease (IBD) is a chronic, life-threatening inflammatory disease affecting the gastroenterological system, which includes Crohn's disease (CD) and ulcerative colitis (UC) [1, 2]. The etiology and the pathogenesis of inflammatory bowel diseases (IBD) are still not completely understood [3]. Studies have confirmed that hyperalgesia caused by IBD was closely related to the hypersensitivity response triggered by sympathetic-sensory coupling in the skin-dorsal root ganglion (DRG) [4, 5]. A previous study illustrated that sympathetic nerve sprouting in DRG was observed in a model of colitis [6]. On the other hand, neurogenic inflammation was the important mechanism of referred pain caused by visceral lesions on body surfaces [7]. The activation and release of mediators, such as substance P (SP) from unmyelinated afferent nerve endings caused venule vasodilation and increased permeability, which led to inflammatory reactions such as plasma extravasation and edema [8, 9]. Thus, regulation of sympathetic-sensory coupling and neurogenic inflammation provides a new idea for the treatment of visceral hyperalgesia.

It has been well established in traditional Chinese medicine that acupoints which have a diagnostic and curative effect on diseases are an important position for the correlation between the meridian and visceral organs [10, 11]. The acupoints are the paresthesias in corresponding parts of the body surface through neurogenic involved responses in the pathological process of the body [12]. Recently, study reported that neurogenic inflammatory sites were found on the dorsal trunk cutaneous of the liver injury rat model, which was matched with locations of acupoints [13]. Meanwhile, electroacupuncture at neurogenic spots reduced bile duct ligation-induced liver injury [13]. Similarly, our previous study found that colitis rats exhibited secondary hyperalgesia, accompanied by sensitization phenomenon in the Zusanli (ST36) acupoints. Electroacupuncture on Zusanli (ST36) acupoints significantly reduced colon lesions and relieved somatic referred pain of colitis rats [14]. However, the underlying mechanism of electroacupuncture at Zusanli (ST36) relieving colitis and pain hypersensitivity has not been studied.

In this study, we explored whether electroacupuncture at Zusanli (ST36) inhibits DRG sympathetic-sensory coupling and surface neurogenic inflammatory response, thereby reducing colon lesions and eliminating referred pain in a colitis rat model. Furthermore, we also investigated the molecular mechanisms of electroacupuncture at Zusanli (ST36) in the treatment of colitis by regulating the DRG sympathetic-sensory coupling and neurogenic inflammation.

## 2. Materials and Methods

### 2.1. Animal Treatment

**Figure 1:**
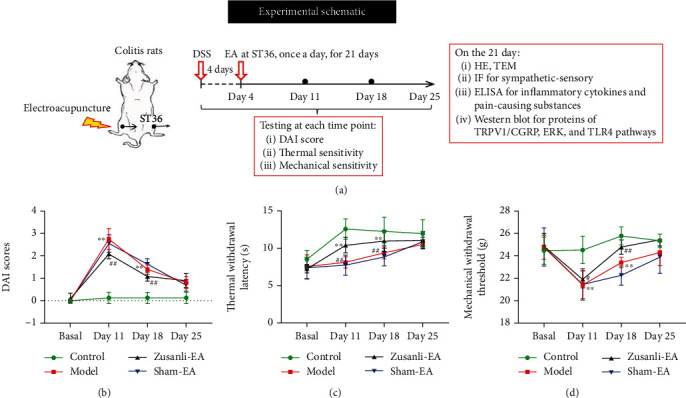
EA attenuated colitis severity and somatic hyperalgesia in DSS-induced colitis rats. (a) Schematic describing experimental design. The rats were gavaged with dextran sulfate sodium (DSS) saline solution for 4 days to establish a colitis rat model. Then, the rats in the Zusanli electroacupuncture (Zusanli-EA) group were treated with EA, once a day, for 21 consecutive days. (b–d) Disease activity index (DAI), thermal withdrawal latency (TWL), and mechanical withdrawal threshold (MWT) were measured at 7 days, 14 days, and 21 days in DSS-induced colitis rats. DAI = (weight loss rate score + stool consistency score + presence of blood in stools score)/3. ^∗∗^*P* < 0.01 vs. control group, ^#^*P* < 0.05 vs. sham-EA group, ^##^*P* < 0.01 vs. sham-EA group. Data represents means ± SD.

All experiments were approved according to the Ethics Committee the Institutional Animal Welfare and Use Committee of the Institute of Acupuncture-Moxibustion, China Academy of Chinese Medicine (no. 20170313). 32 Sprague-Dawley (SD) male rats (SPF grade, 12 weeks, 180-200 g) were purchased from Chengdu Dossy Experimental Animals Co., Ltd. (Chengdu, Sichuan; SCXY (Chuan) 2020-034). The feeding environment was 23 ± 1°C, relative humidity 50 ± 5%, and light/darkness for 12 h circulation. SD rats are allowed to eat and drink freely. The SD rats were randomly divided into 4 groups (*n* = 8), namely, the control group, colitis model group, Zusanli electroacupuncture (Zusanli-EA) group, and sham electroacupuncture (sham-EA) group. For the colitis model group, rats were gavaged with 5% (*w*/*v*) dextran sulfate sodium (DSS) saline solution (MP Biomedicals, Santa Ana, California, USA) for 4 days (50 mL/d) as previously described [15, 16]. The status of rats was monitored using the disease activity index (DAI) [17]. Meanwhile, the control group rats were gavaged an equal volume of saline solution. For the Zusanli-EA group, electroacupuncture was immediately performed under isoflurane inhalation anesthesia after modeling. Rats received electroacupuncture treatment at Zusanli acupoint (ST36, bilateral) using 1.0-inch filigree needles (0.25 mm × 13 mm, Huatuo Brand, depth of about 7 mm). An electroacupuncture treatment device (G6805-2A, Huatuo Brand) was from Suzhou Medical Appliance Factory, China. The electroacupuncture parameter is a dilute wave with 2/100 Hz, the intensity of 1 mA, and performed for 15 minutes, once a day, for 21 consecutive days as previously described [18]. For the sham-EA group, rats were anesthetized by inhalation of 3–4% isoflurane and then received sham electroacupuncture with a pragmatic placebo needle on sham acupoints. The neurological function and DAI score of rats were tested per week. At the end of the 3-week administration, all rats were anesthetized with 1% sodium pentobarbital (50 mg/kg) and euthanized. The colon tissues, serum, ipsilateral lumbar 6 (L6) dorsal root ganglia (DRG), and nearby skin were removed and kept at -80°C for subsequent analysis. The flow of subjects through the experimental procedure is described in Figure 1(a).

### 2.2. Measurement of Thermal Sensitivity

A BME-410C thermal stimulation meter (Tianjin Berne Technology Co., Ltd., Tianjin, China) was used to detect thermal withdrawal latency (TWL) of rat hind paws according to published methods [19]. Briefly, the rats were placed in a hot plate instrument (52°C), and the paw-withdrawal latency is defined as the time since the foot touches the hot plate instrument until the hind paw licking (s).

### 2.3. Measurement of Mechanical Sensitivity

A BME-404 electrical mechanical analgesia tester (Institute of Biomedical Engineering Chinese Academy of Medical Sciences, Tianjin, China) was used to measure the mechanical withdrawal threshold (MWT) of the rat hind paw. Stainless steel filaments (0.6 mm in diameter) were employed to stimulate the plantar surface of the left hind paw with pressure. When a retracted paw response occurs, the force (g) was automatically recorded.

### 2.4. Hematoxylin and Eosin (H&E) Stain

The colon tissues were collected and fixed in 4% paraformaldehyde overnight, processed, and embedded in paraffin. The tissue sections were stained with hematoxylin and eosin (H&E) to observe the degree of the lesion and inflammatory cell infiltration under 10x and 400x magnification optical microscope (Olympus BH2, Tokyo, Japan).

### 2.5. Transmission Electron Microscopy (TEM)

The pathological changes in colon tissues were observed by TEM. Briefly, colon tissues were fixed with 3% glutaraldehyde for 15 min and postfixed with 1% osmium tetroxide for 2 h at 4°C. The colon tissues were then incubated with propanone for 2 h and embedded with Ep812 resin. The blocks were sliced with a Leica EM UC7, and sample sections were stained with uranium acetate-lead citrate. A JEM-1400Flash transmission electron microscopy (JEOL; Tokyo, Japan) was used to examine.

### 2.6. Immunohistochemistry (IHC) Stain

Paraffin blocks were sectioned at 4 *μ*m. IHC stain was performed to detect the 5-hydroxytryptamine (5-HT) expression in skin tissue corresponding to the L6 DRG of mice according to instructions of the IHC protocol. 5-HT antibody was purchased from Beijing Zhongshan Golden Bridge Biotechnology Co., Ltd. (BS-1126R, Beijing, China; 1/100). IHC images were captured using a digital trinocular camera microscope camera system (BA400Digital, Motic Instruments, Inc., Baltimore, MD, USA). Image quantification was performed using Halo software (Indica Labs; Albuquerque, NM).

### 2.7. Immunofluorescence (IF) Stain

Paraffin sections of L6 DRG were dewaxed and hydrated. The sections were incubated in QuickBlock™ Blocking Buffer (Beyotime Biotechnology, Jiangsu, China, P0260) for 30 min at room temperature. Then, the sections were incubated with calcitonin gene-related peptide (CGRP) antibody (bs-0791R, Beijing Bosen Biological Technology Co., Ltd. Beijing, China, 1/100), tyrosine hydroxylase (TH) antibody (ab129991, Abcam; 1/100), or NeuN (ab129991, Abcam; 1/100) antibody at 4°C overnight and washed 3 times with phosphate-buffered saline (PBS, ZLI-9062, Beijing Zhongshan Golden Bridge Biotechnology Co., Ltd., Beijing, China). Then, DAPI (ZLI-9557; Beijing Zhongshan Golden Bridge Biotechnology Co., Ltd., Beijing, China) was added dropwise into the sections for 5 min. The staining was observed under a fluorescence microscope Olyvia (Olympus, Tokyo, Japan) at 100x and 400x magnifications. Image processing was conducted with Image J (National Institutes of Health, Bethesda, MA, USA).

### 2.8. Western Blot Analysis

L6 DRG tissues were treated using RIPA buffer (Signaling Technology, Inc.). The concentration of protein was determined by a BCA kit (Sigma-Aldrich; Merck KGaA). Total protein (30 *μ*g/sample) was separated via 10% SDS-PAGE. And then, the separated proteins are transferred to nitrocellulose membranes. The membranes were blocked with 5% nonfat dried milk overnight at 4°C and incubated with corresponding protein antibodies. Then, the membranes were washed with Tris-buffered saline/0.1% Tween (TBST) and incubated for 1.5 hours with an HRP Goat anti-Rabbit IgG. The bands were visualized using the ECL system (Affinity Biosciences, Cincinnati, Ohio, USA), and *β*-actin was used as an internal control. The net optical density was measured using Quantity One software (Bio-Rad). Antibody information used for Western blot analysis is shown in Table 1.

### 2.9. Detection of the Levels of Inflammatory Cytokines in Serum and Pain-Causing Substances in Skin Tissues

The levels of interleukin (IL)-1*β*, IL-6, tumor necrosis factor (TNF)-*α*, prostaglandin E2 (PGE2), and IL-10 in rat serum and substance P (SP), hyaluronic acid (HA), bradykinin (BK), and prostacyclin (PGI2) in rat skin tissues (corresponding to the L6 DRG) were examined by quantizing enzyme-linked immunosorbent assay (ELISA) kit (ZhuoCai Biological Technology, China) based on the manufacturer's instructions. The absorbance of wells was measured with a microplate reader (SpectraMAX Plus384, USA) at 450 nm wavelength to calculate the sample concentration.

### 2.10. Statistical Analysis

The data were represented as means ± standard deviation. Statistical analysis was performed using SPSS 20.0 (IBM Corp.). One-way analysis of variance (ANOVA) with Tukey's post hoc test and Student's *t*-test was used for statistical analysis. Differences with a *P* < 0.05 were considered to indicate statistically significant.

## 3. Results

### 3.1. EA Attenuated Colitis Severity and Somatic Hyperalgesia in DSS-Induced Colitis Rats

**Figure 2:**
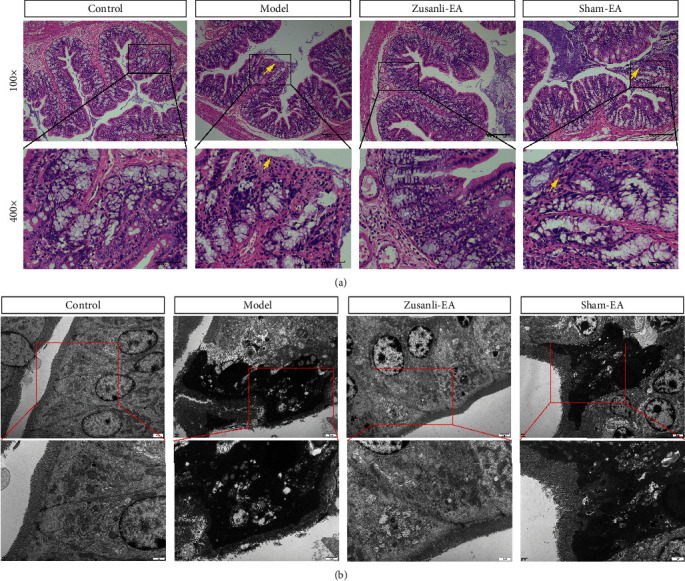
EA improved pathological lesions of L6 DRG in DSS-induced colitis rats. (a) Representative picture of colonic H&E stain. The magnifications are 100x and 400x. The destruction of mucosal layers is indicated by the arrows. (b) Transmission electron microscopy (TEM) micrograph of L6 DRG in DSS-induced colitis rats. The scale bar of the upper panel is 2 *μ*m. The scale bar of the lower panel is 1 *μ*m.

DAI score was undertaken weekly to evaluate the progression of colitis. As compared with the control, the DAI score was significantly increased on day 7 after DSS treatment (Figure 1(b)). EA treatment continuously reduced the DAI score. On days 11 and 14, the DAI score was significantly decreased compared with the sham-EA group (Figure 1(b)). Meanwhile, colitis rats displayed lower TWL and MWT on days 11 and 14, which were dramatically corrected by EA treatment (Figures 1(c) and 1(d)). These results indicated that the therapeutic effect of EA at Zusanli in the early stage of colitis in rats is significant. Furthermore, the results of the H&E stain showed that model group colonic mucosa was severely damaged, with a high number of infiltrating inflammatory cells in comparison to the control group (Figure 2(a)). It was found that the EA group exhibited significantly reduced mucosal injury and infiltration of inflammatory cells compared with the sham-EA group (Figure 2(a)). Meanwhile, we also used TEM to observe the pathological changes of colon tissues. As compared with the control, colitis rats exhibited severe colonic injury marked by severe damage appeared including transmural infiltration of inflammatory cells, necrosis, and destruction of crypts, massive transmural inflammatory cell infiltration, and thickening of the colonic wall (Figure 2(b)). EA treatment significantly improved DSS-induced damage to colon tissues compared with the sham-EA group (Figure 2(b)). These data suggested that EA treatment alleviated DSS-induced colonic injury and hypersensitivity in colitis rats.

### 3.2. EA Attenuated Sympathetic-Sensory Coupling of L6 DRG in DSS-Induced Colitis Rats

**Figure 3:**
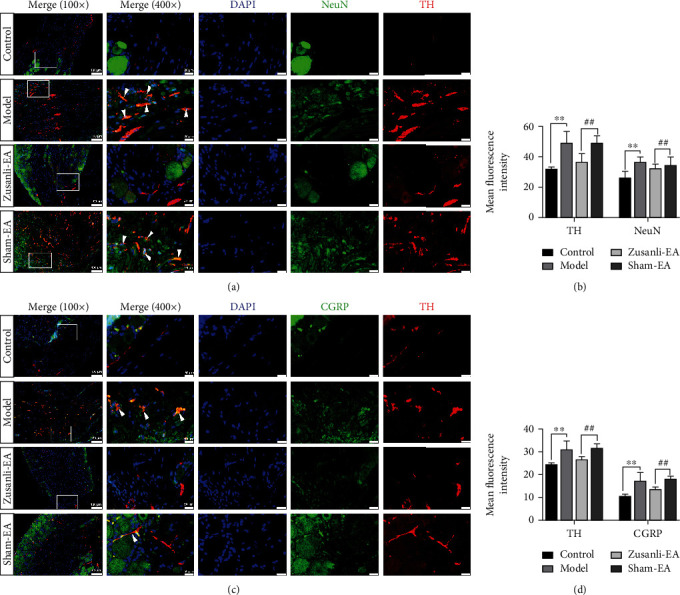
EA attenuated sympathetic-sensory coupling of L6 DRG in DSS-induced colitis rats. (a) TH/NeuN/DAPI immunofluorescence (IF) stain in the L6 DRG. Blue labeling is for DAPI, green labeling is for NeuN, and a red one for TH. (b) Quantitative immunofluorescence (IF) analysis. (c) Immunofluorescence (IF) stain for TH (green), CGRP (red), and DAPI (blue) and colocalization of TH and CGRP in the L6 DRG. (d) Quantification of TH and CGRP immunofluorescence (IF). ^∗∗^*P* < 0.01 vs. control group, ^##^*P* < 0.01 vs. sham-EA group. Data represents means ± SD.

Compared with the control group, the distribution of TH-positive sympathetic fibers in the DRG was increased at 21 d after DSS induction (Figures 3(a) and 3(b)). Meanwhile, the TH-positive sympathetic fibers wrapped around sensory neurons to form sympathetic-sensory coupling (Figures 3(a) and 3(b)). EA treatment decreased the expression of TH and inhibited sympathetic fibers from wrapping around sensory neurons (Figure 3(a)). In addition, sensory nerve fibers were labeled with CGRP, which was a factor in neurogenic inflammatory responses. As shown in Figure 3(b), the density of CGRP-positive sensory nerve fibers and TH-positive sympathetic fibers were both increased in L6 DRG of colitis rats (Figures 3(c) and 3(d)). Compared with the sham-EA group, EA treatment suppressed sympathetic nerve sprouting in L6 DRG of colitis rats (Figures 3(c) and 3(d)). These findings confirmed that DSS induction promoted the sprouting of sympathetic nerve fibers which corresponded to the increased sympathetic nerve activity. EA intervention ameliorated these changes.

### 3.3. EA Decreased Neurogenic Inflammatory on Body Surfaces in DSS-Induced Colitis Rats

**Figure 4:**
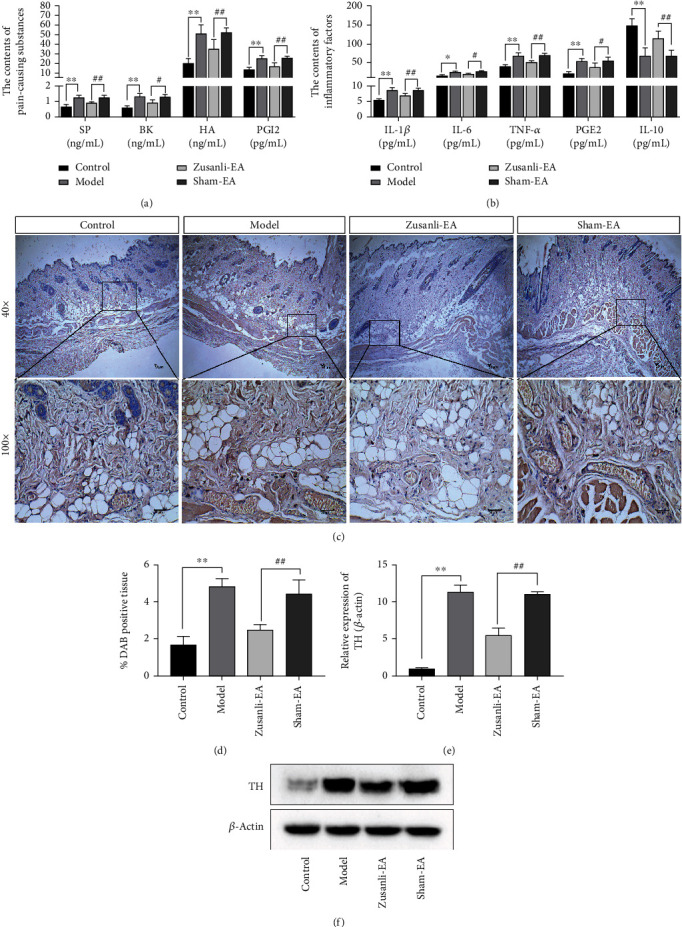
EA decreased neurogenic inflammatory on body surfaces in DSS-induced colitis rats. (a) The contents of pain-causing substances SP, BK, HA, and PGI2 in the rat skin tissues (corresponding to the L6 DRG) were examined by enzyme-linked immunosorbent assay (ELISA). (b) The contents of inflammatory factors IL-1*β*, IL-6, TNF-*α*, PGE2, and IL-10 in rat serum were tested by enzyme-linked immunosorbent assay (ELISA). (c) Immunohistochemistry (IHC) for 5-HT expression in the rat skin tissues (corresponding to the L6 DRG). (d) Quantification of positive immunohistochemical (IHC) stain. (e, f) The expression of TH in L6 DRG of colitis rats was tested by Western blot analysis. ^∗^*P* < 0.05 vs. control group, ^∗∗^*P* < 0.01 vs. control group, ^#^*P* < 0.05 vs. sham-EA group, ^##^*P* < 0.01 vs. sham-EA group. Data represents means ± SD.

To evaluate DSS-associated neurogenic inflammatory on body surfaces, we performed an ELISA assay. Three weeks after DSS induction, the expression levels of neurogenic inflammatory response-related inflammatory and pain-causing substances SP, HA, BK, and PGI2 in skin tissues were significantly increased compared with the control group (Figure 4(a)). EA treatment decreased the expression of SP, HA, BK, and PGI2 compared with the sham-EA group (Figure 4(a)). Meanwhile, the secretion of proinflammatory factors IL-1*β*, IL-6, TNF-*α*, and PGE2 was enhanced, as well as the levels of anti-inflammatory factor IL-10 were reduced in the serum of colitis rats, which was all reversed by EA treatment (Figure 4(b)). IHC stain showed that DSS induction promoted neurotransmitter 5-HT expression in skin tissues, which was also eliminated by EA treatment (Figures 4(c) and 4(d)). Meanwhile, TH was reported to be a key enzyme that regulates neurotransmitters in nerve cells [20]. The increased TH expression induced by DSS was dramatically blocked by EA treatment in L6 DRG of colitis rats (Figures 4(e) and 4(f)).

### 3.4. EA Inhibited TRPV1/CGRP, ERK, and TLR4 Signaling Pathways in DSS-Induced Colitis Rats

**Figure 5:**
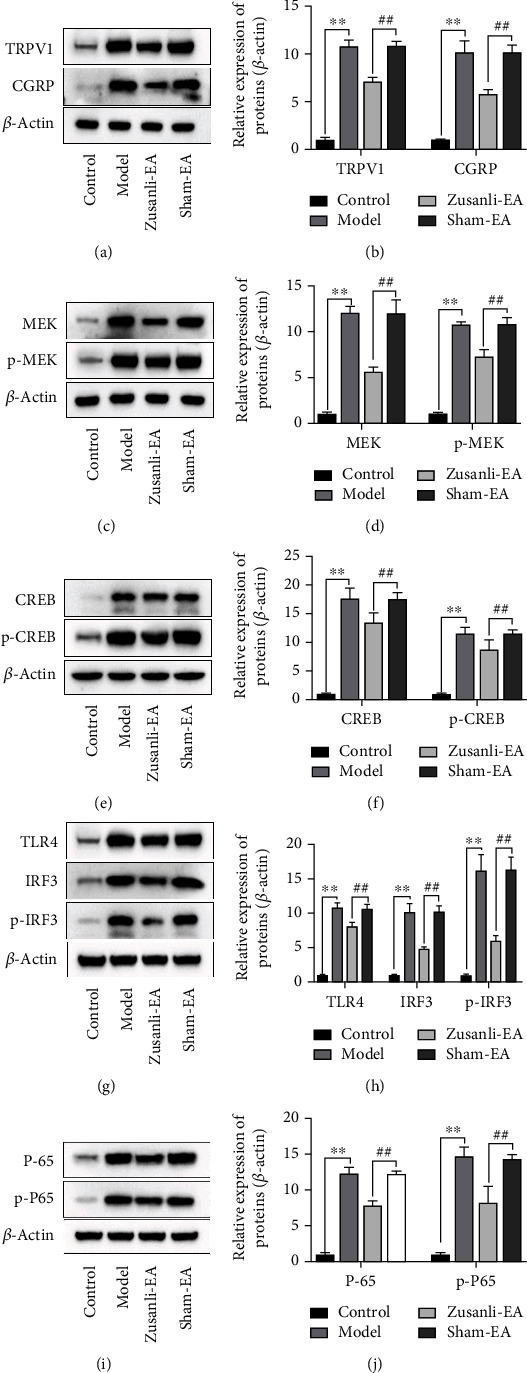
EA inhibited TRPV1/CGRP, ERK, and TLR4 signaling pathways in DSS-induced colitis rats. Representative Western blot analysis and quantification of densitometries of Western blot band for the expression of TRPV1 and CGRP (a, b), MEK and p-MEK (c, d), CREB and p-CREB (e, f), TLR4, IRF3, and p-IRF3 (g, h), and P-65 and p-P65 (i, j) in L6 DRG of DSS-induced colitis rats. *β*-Actin was used as a loading control. ^∗∗^*P* < 0.01 vs. control group, ^##^*P* < 0.01 vs. sham-EA group. Data represents means ± SD.

To elucidate the underlying cellular mechanism of EA stimulation, we analyzed whether EA at ST36 augmented TRPV1/CGRP, ERK, and TLR4 signaling pathway activation, which were known to be a crucial role in the neurogenic inflammatory. We found that DSS induction resulted in the activation of TRPV1/CGRP, ERK, and TLR4 signaling pathways, characterized by the increased expression of TRPV1, CGRP, MEK, p-MEK, CREB, p-CREB, TLR4, IRF3, p-IRF3, P-65, and p-P65. EA stimulation significantly inhibited this process, whereas EA at nonacupoint did not produce a significant improvement (Figures 5(a)–5(j)).

## 4. Discussion

It is well known that referred somatic pain was caused by activation of primary nociceptive afferents by visceral lesions stimuli [21]. Referred somatic pain was accompanied by secondary hyperalgesia, reflex muscle spasms, deep tenderness, and autonomic hyperactivity [22, 23]. Studies have found that the sympathetic nervous system was closely related to hyperalgesia due to visceral lesions [24–26]. Sympathetic nerve sprouts in the DRG by coupling around neurons to form sympathetic-sensory coupling were found in animal models of pathological conditions. Referred somatic pain and peak sympathetic sprouting were observed in the neuropathic pain model of the cuff and spared nerve injury (SNI) in the sciatic territory [27]. A previous study found that nerve growth factor (NGF) released from the sprouted sympathetic fibers in the synovial membrane and upper dermis contributed to the pain-related behavior associated with arthritis [28]. It has been shown that jaw pain due to myocardial ischemia could be explained by the convergence of cardiac visceral afferent fibers with spinothalamic tract (STT) neurons [29]. In a rat model of trinitrobenzene sulfonic acid- (TNBS-) induced colitis, sympathetic nerve fiber sprout was found in the DRG of the lumbosacral segment (L6, S1), manifested by tyrosine hydroxylase- (TH-) positive nerve fibers increased [30]. Dextran sulfate sodium (DSS) treatment caused mechanical hypersensitivity in the abdominal and facial skin of colitis mice by increasing TRPA1 expression in cultured DRG neurons and selectively enhanced currents evoked by the TRPA1 agonist [31]. Importantly, in the current study, we detected that DSS induced colon tissue lesions and somatic hyperalgesia in rats. Moreover, DSS treatment increased sprouting of sympathetic fibers in L6 DRG into the sensory ganglia.

On the other hand, neurogenic inflammation was an important part of the pathogenesis of referred somatic pain. Primary afferent nociceptive neurons released neuropeptides to the periphery, leading to mast cell degranulation and the release of biologically active substances that produce pain and/or inflammation such as CGRP, SP, 5-HT, HA, BK, and PGI2, which induced neurogenic inflammation characterized by vasodilatation, protein extravasation, and leukocyte migration [32, 33]. In the inflammatory pain model, PGI2 was involved in pain transmission at the spinal cord [34]. 5-HT participated in the mediation of joint pain in experimental arthritis by exciting and sensitizing the medial articular afferent nerve [35]. A previous report indicated that the blockade of receptor channels such as TRPV1 and TRPA1 on nociceptive sensory neurons was shown to attenuate experimental colitis by suppressing the release of GRP and SP [36]. Furthermore, HA sensitized the nociceptor TRPV1 in mouse nociceptive dorsal root ganglion neurons and was known to contribute to relieving visceral hypersensitivity, symptoms, and abdominal pain in IBD patients [37]. Our results demonstrated that DSS colitis upregulated SP, HA, BK, PGI2, and 5-HT expression in the skin and increased TRPV1, TRPA1, and TH in L6 DRG, implying that DSS promoted surface neurogenic inflammation evoked by sympathetic-sensory coupling in skin-DRG.

EA, as a traditional therapeutic method, has been used to treat IBD and hyperalgesia in China. EA treatment at Zusanli (ST36) attenuated the macroscopic damage and the myeloperoxidase activity of colonic samples [38]. Furthermore, EA at Zusanli (ST36) and Guanyuan (CV4) activated microglia in hippocampus CA1 and CA3 regions of DSS-induced colitis mice [39]. Interestingly, EA at Zusanli (ST36) and Shangjuxu (ST37) significantly reduced the severity of colonic inflammation, as well as the visceral hypersensitivity and referral hind paw hyperalgesia in colitis rats by increasing [40]. EA at Zusanli (ST36) eliminated the expression and activation of mast cells and improved visceral hypersensitivity in experimental colitis [41]. The mechanism may be via inhibiting of NGF/TrkA/TRPV1 peripheral afferent pathway triggered by the mast cells [41]. We found that EA at Zusanli (ST36) improved the pathological state of the colon tissues and referred somatic pain in a colitis rat model, which was related to the inhibiting of sensory-sympathetic coupling in L6 DRG and neurogenic inflammation in the skin.

As is well known, TLR4 initiated downstream genes such as NF-KB and IRF3 and activated the expression of inflammatory factors such as IL-1*β*, IL-6, and TNF-a, thereby inducing inflammatory responses and pain-related hypersensitivity. Increased expression of TLR4, p-p65, TNF-*α*, and IL-1*β* in (L4/L5) DRGs was observed in a postoperative pain model [42]. Importantly, TNBS treatment enhanced TLR4 and TRPV1 coexpression in primary afferents including the trigeminal sensory neurons and DGR neurons of colitis mice [43]. Additionally, suppressing the synthesis of ERK in DRG has proven effective to alleviate hyperalgesia. The deactivation of the MEK/ERK pathway in the DRG of chronic constriction injury rats alleviated neuropathic pain development [44]. H_2_O_2_-induced hyperalgesia was related to increased phosphorylation of ERK in neurons of DRG [45]. A recent study found that in a rat model of colitis, the activation of ERK5 mediated BDNF upregulation in the DRG primary afferent neurons [46]. In this research, we demonstrated that DSS induction activated TRPV1/CGRP, ERK, and TLR4 signaling pathways, which were significantly offset by EA at Zusanli (ST36).

In conclusion, EA at Zusanli (ST36) relieved hyperalgesia induced by colitis via the inhibition of surface neurogenic inflammation and sympathetic sprouting into the DRG, which were mediated by TRPV1/CGRP, ERK, and TLR4 signaling pathway deactivation. EA at Zusanli (ST36) may be an effective treatment for referred somatic pain in UC patients.

## Figures and Tables

**Table 1:** Antibodies used for Western blot analysis.

Antibody	Type	Dilution	Source
CGRP	Rabbit polyclonal	1/1000	A5542, ABclonal, Wuhan, China
CREB	Rabbit polyclonal	1/500	A10826, ABclonal, Wuhan, China
ERK	Rabbit polyclonal	1/1000	A4782, ABclonal, Wuhan, China
IRF3	Rabbit polyclonal	1/500	A11118, ABclonal, Wuhan, China
MEK	Rabbit polyclonal	1/1000	A12687, ABclonal, Wuhan, China
P65	Rabbit polyclonal	1/500	A19653, ABclonal, Wuhan, China
p-CREB	Rabbit polyclonal	1/200	AP0019, ABclonal, Wuhan, China
p-ERK	Rabbit polyclonal	1/1000	#4370, Cell Signaling Technologies, Danvers, MA, USA
p-IRF3	Rabbit polyclonal	1/1000	AP0632, ABclonal, Wuhan, China
p-MEK	Rabbit polyclonal	1/1000	AP1021, ABclonal, Wuhan, China
p-p65	Rabbit polyclonal	1/1000	#3033, Cell Signaling Technologies, Danvers, MA, USA
TH	Rabbit polyclonal	1/500	A0028, ABclonal, Wuhan, China
TLR4	Rabbit polyclonal	1/500	A5258, ABclonal, Wuhan, China
TRPV1	Rabbit polyclonal	1/500	A8564, ABclonal, Wuhan, China
*β*-Actin	Rabbit polyclonal	1/50000	AC026, ABclonal, Wuhan, China
IgG (H + L)	Goat anti-rabbit	1/2000	ab6721, Abcam, Cambridge, UK

## Data Availability

The datasets used or analyzed during the current study are available from the corresponding author upon reasonable request.
